# AMPlify: attentive deep learning model for discovery of novel antimicrobial peptides effective against WHO priority pathogens

**DOI:** 10.1186/s12864-022-08310-4

**Published:** 2022-01-25

**Authors:** Chenkai Li, Darcy Sutherland, S. Austin Hammond, Chen Yang, Figali Taho, Lauren Bergman, Simon Houston, René L. Warren, Titus Wong, Linda M. N. Hoang, Caroline E. Cameron, Caren C. Helbing, Inanc Birol

**Affiliations:** 1 Canada’s Michael Smith Genome Sciences Centre, BC Cancer Agency, Vancouver, BC V5Z 4S6 Canada; 2 Bioinformatics Graduate Program, University of British Columbia, Vancouver, BC V6T 1Z4 Canada; 3 Public Health Laboratory, British Columbia Centre for Disease Control, Vancouver, BC V5Z 4R4 Canada; 4 Department of Pathology and Laboratory Medicine, University of British Columbia, Vancouver, BC V6T 1Z4 Canada; 5 Department of Biochemistry and Microbiology, University of Victoria, Victoria, BC V8P 5C3 Canada; 6 Medical Microbiology Laboratory, Vancouver General Hospital, Vancouver, BC V5Z 1M9 Canada; 7 Division of Infectious Diseases, Department of Medicine, University of Washington, Seattle, WA 98195 USA; 8 Department of Medical Genetics, University of British Columbia, Vancouver, BC V6H 3N1 Canada

**Keywords:** Antimicrobial peptide, Deep learning, Attention mechanism

## Abstract

**Background:**

Antibiotic resistance is a growing global health concern prompting researchers to seek alternatives to conventional antibiotics. Antimicrobial peptides (AMPs) are attracting attention again as therapeutic agents with promising utility in this domain, and using in silico methods to discover novel AMPs is a strategy that is gaining interest. Such methods can sift through large volumes of candidate sequences and reduce lab screening costs.

**Results:**

Here we introduce AMPlify, an attentive deep learning model for AMP prediction, and demonstrate its utility in prioritizing peptide sequences derived from the *Rana [Lithobates] catesbeiana* (bullfrog) genome. We tested the bioactivity of our predicted peptides against a panel of bacterial species, including representatives from the World Health Organization’s priority pathogens list. Four of our novel AMPs were active against multiple species of bacteria, including a multi-drug resistant isolate of carbapenemase-producing *Escherichia coli*.

**Conclusions:**

We demonstrate the utility of deep learning based tools like AMPlify in our fight against antibiotic resistance. We expect such tools to play a significant role in discovering novel candidates of peptide-based alternatives to classical antibiotics.

**Supplementary Information:**

The online version contains supplementary material available at 10.1186/s12864-022-08310-4.

## Background

As reported by the World Health Organization (WHO), the decreasing effectiveness of antibiotics and other antimicrobial agents indicates the world is at a risk of entering a “post-antibiotic era” [1]. To counter this threat, new drugs or effective substitutes for conventional antibiotics are urgently needed. Antimicrobial peptides (AMPs) are one such alternative. AMPs are host defense molecules produced by all forms of life, including multicellular organisms as part of their innate immunity against microbes. Within their respective hosts, eukaryotic AMPs have co-evolved with microorganisms to serve as a defense against bacterial [2], fungal [3] and even viral infections [4]. Unlike most conventional antibiotics, which have specific functional or structural targets, AMPs act directly on the microorganisms, often causing cell lysis, or modulate the host immunity to enhance defense against microorganisms [5]. Also, they act faster than conventional antibiotics [6], have a narrower active concentration window for killing [7], and do not typically damage the DNA of their targets [8, 9]. As a result, they do not induce resistance to the extent that is observed with conventional antibiotics [10]. Nevertheless, if bacteria are exposed to AMPs for extended periods of time, they can and do develop resistance even to peptide-based drugs including the last resort and life-saving drug, colistin [10, 11]. Hence, fast and accurate methods would be valuable tools to discover and design effective AMPs to enhance our repertoire of alternative therapeutics.

Direct, large scale discovery of novel AMPs through wet lab screening is time-consuming, labor-intensive and costly [12]. For these reasons, various computational models have been developed over the last few years [12] to streamline in silico AMP prediction. Despite the rapid progress in the field, currently available models still have substantial room for improvement.

The AMP prediction module in the Collection of Antimicrobial Peptides (CAMP) database [13] includes four different models: random forest, support vector machine, discriminant analysis, and a single-hidden-layer feed-forward neural network with 64 designed features [14]. The iAMP-2L online server adopts fuzzy *K*-nearest neighbor algorithm, taking pseudo amino acid compositions (PseAAC) with five physicochemical properties as input features to predict AMPs as well as their potential microorganism targets [15]. The iAMPpred online server for AMP prediction and classification is based on support vector machine and uses PseAAC with compositional, physicochemical, and structural features [16]. All three of these tools employ conventional machine learning methods and rely on pre-designed features, requiring prior expertise in AMP structure and mechanism for effective engineering.

Alternatively, deep learning methods can automatically learn high-level features and usually outperform conventional methods in many bioinformatics tasks [17]. Recently, few teams developed deep learning models for the AMP prediction task. Youmans and co-workers demonstrated the feasibility of using a bidirectional long short-term memory [18–20] (Bi-LSTM) recurrent neural network (RNN) for AMP prediction [21], but the authors do not offer any public code or tool that implements their model. The Deep-AmPEP30 online server applies a convolutional neural network (CNN) for AMP prediction [22], though the tool is restricted to working with short peptides up to 30 amino acids (aa) in length. The Deep-ABPpred online server adopts Bi-LSTM with word2vec [23], also for short (≤ 30 aa) peptides [24]. The Bi-LSTM model from Wang and co-workers is designed for even shorter peptides (≤ 20 aa) and specializes to predicting AMPs against *Escherichia coli* [25]. They also provide a workflow for designing novel AMPs. Veltri and co-workers introduced a deep neural network classifier with embedding, convolutional, max pooling, and long short-term memory (LSTM) recurrent layers which is available as an online server, AMP Scanner Vr.2, as its user interface [26]. AMP Scanner Vr.2 is the only tool in the deep learning category that does not have a strong limitation in input sequence lengths; it can handle sequences up to 200 aa.

While AMP Scanner Vr.2 outperforms the conventional machine learning methods cited above, we note that its neural network architecture is not designed for extracting long-range information along peptide sequences. Common deep learning methods for sequence classification include recurrent neural networks (RNNs) and convolutional neural networks (CNNs), as employed in combination by AMP Scanner Vr.2. RNNs can learn remote dependencies inside a sequence, but suffer from vanishing gradients [27]. Similarly, while CNNs can extract local information well, it ignores long-range dependencies [28].

Recently, deep neural networks with attention mechanisms have gained interest, notably for natural language processing [29–31] and computer vision [32] applications. Attention mechanisms, as the name suggests, are inspired by our brains’ ability to prioritize segments of information when processing textual or visual input. In sequence analysis, attention mechanisms are modeled by weights assigned to different positions in a sequence. These weights amplify or attenuate information from a given position to help encode the global information of the sequence.

**Fig. 1:**
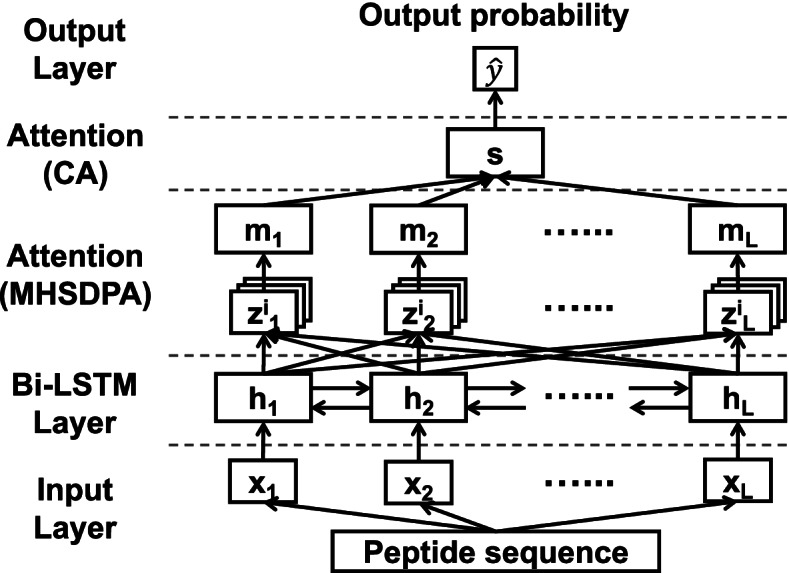
Model architecture of AMPlify. Residues of a peptide sequence are one-hot encoded and passed to three hidden layers in order: the bidirectional long short-term memory (Bi-LSTM) layer, the multi-head scaled dot-product attention (MHSDPA) layer and the context attention (CA) layer. The output layer generates the probability that the input sequence is an AMP

Here, we introduce AMPlify, an attentive deep learning model that improves in silico AMP prediction by applying two types of attention mechanisms layered on a bidirectional long short-term memory [18–20] (Bi-LSTM) layer (Fig. 1). The Bi-LSTM layer in the model, as a variant of RNN, encodes positional information from the input sequence in a recurrent manner. Subsequently, the multi-head scaled dot-product attention [30] (MHSDPA) layer computes a refined representation of the sequence using multiple weight vectors. The last hidden layer of context attention [31] (CA) generates a single summary vector using weighted average, learning contextual information gained from the previous layer. The AMPlify model is trained on a set of known AMPs and a select list of non-AMP sequences, and adopts ensemble learning to further improve its performance. To the best of our knowledge, AMPlify is the first machine learning application that applies attention mechanisms for in silico AMP prediction. We note that non-standard amino acids are not taken into consideration in this study, and we mainly focus on AMPs from multicellular organisms for discovery.

To illustrate the utility of our model, a discovery pipeline based on AMPlify was used to mine the AMP-rich North American bullfrog (*Rana [Lithobates] catesbeiana*) genome for novel natural AMPs. Previously, the North American bullfrog has been described as a rich source for natural AMPs, yielding potent classes of bioactive molecules such as ranateurins, ranacyclins, temporins, and palustrins [33, 34]. In our tests, AMPlify successfully identified these previously reported AMPs, along with four novel AMPs with biological activity in vitro.

The WHO has a published list of priority pathogens for which new antibiotics are urgently needed [35]. This list includes bacterial species that are increasingly resistant to multiple antibiotics*.* We tested the efficacy of our discovered, putative AMPs against selected Priority Pathogens, including: 1) *Pseudomonas aeruginosa* and *Escherichia coli* strains, including a multi-drug resistant (MDR) carbapenemase-producing (CPO) strain of *E. coli* reflective of WHO’s “Priority 1” pathogens; and 2) a *Staphylococcus aureus* strain reflective of WHO’s “Priority 2” methicillin-resistant (MRSA) and vancomycin-resistant (VRSA) strains. A *Streptococcus pyogenes* strain was included as an additional Gram-positive bacterial species that causes human disease, while this bacterial species has demonstrated antibiotic resistance in some earlier works [36].

In our tests, four of the 16 novel AMPs discovered show considerable antimicrobial potency against one or more of the organisms examined, including the clinical MDR isolate of CPO *E. coli.* These results highlight the potential of AMPlify to accelerate AMP discovery, the first step towards facilitating peptide-based therapeutics.

## Results

### Evaluation of model architecture

To demonstrate the effectiveness of each component within our model, we evaluated the model architecture starting from a single Bi-LSTM layer and then gradually adding attention layers over it. Supplementary Table S1 summarizes the results of our ablation study, comparing different model architectures using stratified 5-fold cross-validation on the training set with regard to five different measures of (1) accuracy, (2) sensitivity, (3) specificity, (4) F1 score, and (5) area under the receiver operating characteristic curve (AUROC). The first section of the table compares the performance of the complete architecture of AMPlify, with and without ensemble learning, with simpler variations, which include fewer hidden layers. The architecture of the only deep learning based comparator, AMP Scanner Vr.2, was cross-validated on our training set for comparison using two different stopping settings: the optimal fixed number of epochs as stated in their manuscript [26] and early stopping as described in this paper (Supplementary Table S1, second section). Although overall performance of AMP Scanner Vr.2 is not strongly influenced by early stopping, it does lead to smaller performance variability as measured by standard deviation values in tests, indicating that the model trained using early stopping is more robust than using a default of 10 epochs.

By adding a single CA layer atop the Bi-LSTM layer, the model performs similarly to AMP Scanner Vr.2 based on cross-validation results, with differences smaller than 1% in all metrics except specificity (< 1.4%). After inserting an MHSDPA layer in the middle, the cross-validation results for our model reach 91.70% in accuracy, 91.40% in sensitivity, 92.00% in specificity, 91.68% in F1 score, and 96.92% in AUROC – an overall improvement compared with the architecture without this layer. This indicates that the attention layer learns discriminating features of sequences processed by the Bi-LSTM layer. We note that the final AMPlify architecture already outperforms the AMP Scanner Vr.2 architecture in all metrics in our cross-validation tests. After applying ensemble learning to the proposed architecture, the cross-validation performance is further improved to 92.79% for accuracy, 92.12% for sensitivity, 93.47% for specificity, 92.74% for F1 score and 97.44% for AUROC.

To test whether the improvement of our model is statistically significant, we performed paired Student t-tests based on cross-validation results. These tests indicate statistically significant increase in performance of AMPlify over AMP Scanner Vr.2 (early stopped) with regard to all five metrics (*p* < 0.05). The better performance of AMPlify without ensemble learning (i.e. Bi-LSTM+MHSDPA+CA) over the simple Bi-LSTM model is also statistically significant in all metrics (*p* < 0.05), suggesting that the attention layers play an important role in the model’s performance.

Further, we cross-validated AMPlify on the dataset provided by AMP Scanner Vr.2 and observed that the deep neural network architecture chosen in AMPlify is overall better for the AMP prediction task compared with the architecture of AMP Scanner Vr.2 (Supplementary Note S1, Supplementary Table S2).

### Comparison with state-of-the-art methods

With the set of hyperparameters tuned through stratified 5-fold cross-validation, the final model of AMPlify was trained using the entire training set, with each of the five single sub-models trained on five different subsets. Here, single sub-model refers to the model with full architecture (Bi-LSTM+MHSDPA+CA) before ensemble learning. AMPlify, along with its single sub-models, were compared on our test set with three other state-of-the-art tools: iAMP-2L [15], iAMPpred [16] and AMP Scanner Vr.2 [26] (Table 1). All the tools were evaluated with their original trained models reported. In this list of comparators, AMP Scanner Vr.2 could be trained using third party datasets through a utility provided by the authors (personal communication with Daniel Veltri), and was re-trained on our training set with two different stopping conditions, as previously stated.

**Table 1:** Performance comparison among different tools on the test set. Performance of different tools are presented with five metrics in percentage: accuracy (acc), sensitivity (sens), specificity (spec), F1 score (F1) and area under the receiver operating characteristic curve (AUROC)

Tool	Model	Acc	Sens	Spec	F1	AUROC
iAMPpred	original^a^	74.01	87.90	60.12	77.18	80.70
iAMP-2L	original^a^	77.96	88.26	67.66	80.02	–
AMP Scanner Vr.2	original^a^	78.50	90.66	66.35	80.83	88.33
	re-trained, 10 epochs^b^	90.66	91.14	90.18	90.70	97.40
	re-trained, early stopped^c^	91.20	90.42	91.98	91.13	97.03
AMPlify	single sub-model 1	92.40	90.90	93.89	92.28	97.54
	single sub-model 2	91.98	91.02	92.93	91.90	97.40
	single sub-model 3	92.51	92.69	92.34	92.53	97.82
	single sub-model 4	92.10	90.90	93.29	92.00	97.27
	single sub-model 5	92.57	92.57	92.57	92.57	97.98
	**ensemble**	**93.71**	**92.93**	**94.49**	**93.66**	**98.37**

^a^Models presented in the referenced papers are available through online servers

^b^The best hyperparameter as stated in the referenced paper

^c^The optimal number of training epochs determined by early stopping is 16

**Fig. 2:**
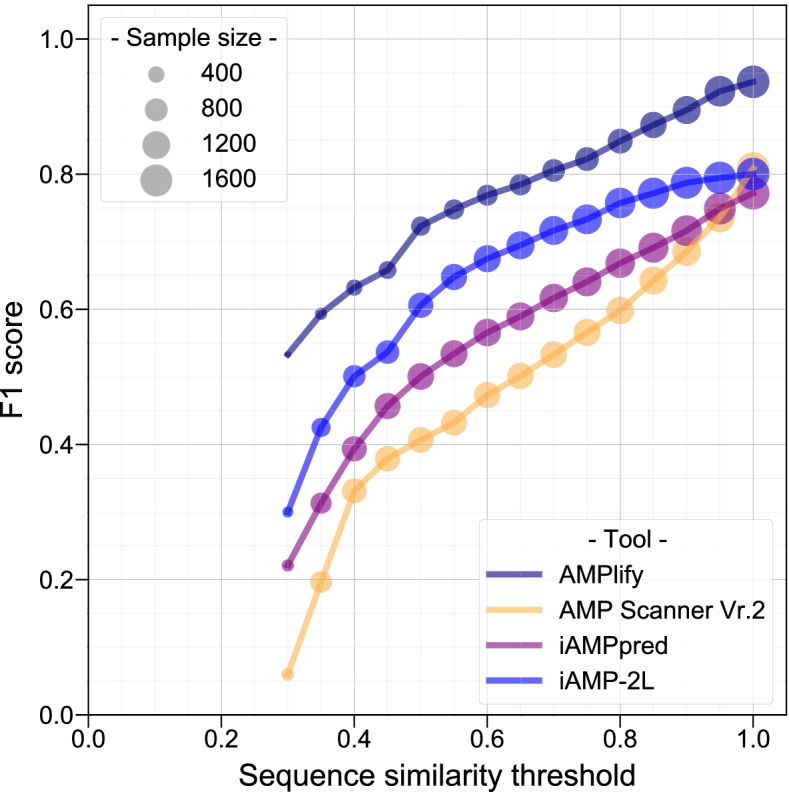
Performance comparison of different AMP prediction tools based on the test sequence similarities to their corresponding training sets. F1 scores of AMP prediction tools were calculated on test subsets based on similarities to sequences in the training sets. All the AMP/non-AMP test subsets were derived from the AMPlify test data, with subsets containing 10 or fewer sequences removed. The size of the round makers indicates the number of sequences remaining in the test subset given the similarity threshold

Among the original models of the three comparators, AMP Scanner Vr.2 performs the best on our data in general, except for its specificity, which is 1.31% lower than iAMP-2L. The accuracy, specificity, F1 score, and AUROC of AMP Scanner Vr.2 were all improved after re-training, with only small changes in sensitivity (< 0.5%). Still, in our benchmarks AMPlify outperforms the comparators tested, including the two re-trained versions of AMP Scanner Vr.2. AMPlify achieves the highest accuracy (93.71%), F1 score (93.66%) and AUROC (98.37%), improving upon the performance of the next-best, the re-trained versions of AMP Scanner Vr.2, by 2.51, 2.53 and 0.97% respectively. AMPlify also shows the highest sensitivity (92.93%) and specificity (94.49%) in our tests, suggesting that the model can concurrently reduce false negative and false positive predictions. We have also analyzed the performance of different tools by stratifying the test set based on sequence similarities to their training sets, again showing how AMPlify performs favourably across this spectrum (Fig. 2, Supplementary Note S2).

Further, all five single sub-models of AMPlify yield favourable performance in accuracy (91.98–92.57%), specificity (92.34–93.89%) and F1 score (91.90–92.57%), despite each single sub-model being trained on 80% of the entire training set (see 9). The sensitivity values of the five single sub-models range from 90.90 to 92.69%, with two of them being better than the performance of all comparators, while the remaining three being slightly lower than the performance of the re-trained, 10 epochs model of AMP Scanner Vr.2 (< 0.25%). Still, the lower standard deviation values from cross-validation analysis indicate that those single sub-models of AMPlify are more robust compared with the re-trained, 10 epochs model of AMP Scanner Vr.2 (Supplementary Table S1). Similarly, our single sub-models score higher than the comparators in AUROC, except one of them being on par with the best AMP Scanner Vr.2 model and another scoring lower by 0.13%. The specificity values of the original models of the three comparators are relatively low (< 70%), likely due to their less stringent selection criteria when building their non-AMP sets. The specificity values of AMP Scanner Vr.2 improved substantially after being re-trained on our training set (90.18% or 91.98%, depending on the number of epochs trained, Table 1). We have also conducted a cross-comparison of AMPlify with AMP Scanner Vr.2, re-training our tool on the dataset provided by the AMP Scanner Vr.2 publication [26], illustrating the improved learning capability of our chosen architecture for the AMP prediction task (Supplementary Note S1, Supplementary Table S3, Supplementary Fig. S1).

**Fig. 3:**
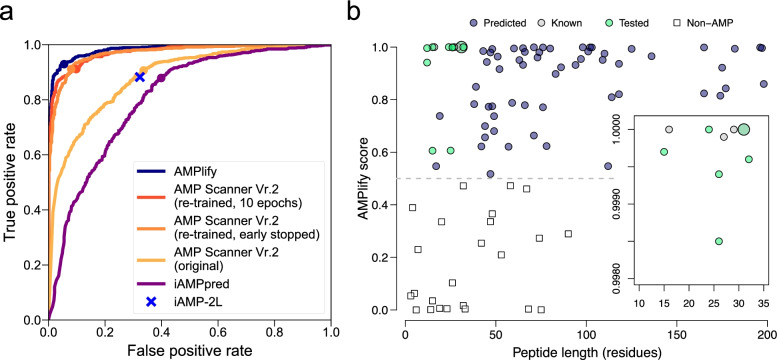
Visualization of AMPlify model performance and the AMP discovery pipeline application results. **a** Receiver operating characteristic (ROC) curves of AMPlify and comparators are plotted, with round dots marking the performance at the threshold of 0.5. The iAMP-2L online server only output labels of AMP/non-AMP without the corresponding probabilities, so it appears as a single point on the plot. **b** AMPlify prediction scores against peptide lengths of 101 sequences analyzed by AMPlify. The grey dotted line represents the score threshold of 0.5 used to distinguish AMPs from non-AMPs. Inset shows amplified view of the upper left region of the plot to enhance visualization of the majority of the selected sequences

For a comparison of the classification performance of each tool with regard to different classification thresholds, Fig. 3a presents a series of receiver operating characteristic (ROC) curves for the models compared. The AUROC results shown in Table 1 correspond to these ROC curves. Note that the iAMP-2L online server does not allow for parameterization, hence the tool is represented by a single data point and no AUROC value. The ROC curves indicate that AMPlify is Pareto-optimal in our tests for any classification threshold.

### AMP discovery

Previous studies have shown that the skin secretions of amphibians are rich in AMPs, which help the animals prevent infection by harmful microorganisms [37]. For this reason, mining the genomes of various frog species for novel AMPs is an attractive proposition. To demonstrate AMPlify’s practical application, it was embedded into a bioinformatics pipeline to find novel AMPs from the North American bullfrog (*Rana [Lithobates] catesbeiana*) genome [33, 34]. For antimicrobial susceptibility testing (AST), we focus on cationic AMPs acting directly on biological membranes, the activities of which can be directly observed in vitro. Most amphibian AMP precursors possess highly conserved N-terminal prepro regions and hypervariable C-terminal antimicrobial domains [37]. The prepro regions usually end with a lysine-arginine signal for cleavage to produce bioactive AMPs [37]. Based on this, we identified candidate precursors from the bullfrog genome using homology search and genome annotation tools. We then derived candidate mature sequences from those precursors to use as input for AMPlify (see 9 for pipeline details). This resulted in 101 candidate mature sequences, which we fed into AMPlify, predicting 75 of them to be putative AMPs. We selected peptides between five to 35 amino acids in length with a positive charge for further analysis, yielding a final list of 16 peptides (Table 2), five of which were previously reported sequences [34, 38, 39]. The remaining 11 peptides were synthesized and evaluated in vitro. The UpSet plot in Supplementary Fig. S2 summarizes the results obtained by applying different combinations of the aforementioned three filters (AMPlify prediction score, length, and charge) to the 101 candidate mature sequences. Figure 3b shows a visualization of AMPlify prediction results for the 101 candidate mature sequences.

**Table 2:** Putative and reported AMP sequences discovered from *Rana [Lithobates] catesbeiana*. Genomic and transcriptomic resources from *Rana [Lithobates] catesbeiana* [33] were mined using the AMP discovery pipeline based on AMPlify. Top-scoring peptide sequences were selected for synthesis and validation in vitro

Peptide Name	Sequence	# aa	Net Charge^a^	MW (Da)	AMPlify Score
**RaCa-1**	GLLDIIKTTGKDFAVKILDNLKCKLAGGCPP	31	2	3242.93	1.0000
**RaCa-2**	FFPIIARLAAKVIPSLVCAVTKKC	24	4	2589.28	1.0000
Ranatuerin-2PRc*	AFLSTVKNTLTNVAGTMIDTFKCKITGVC	29	2	3077.66	1.0000
Temporin-1Cb*^+^	FLFPLITSFLSKFLGK	16	2	1858.30	1.0000
Palustrin-Ca*	GFLDIIKDTGKEFAVKILNNLKCKLAGGCPP	31	2	3303.97	1.0000
Ranatuerin-2RC*	GLFLDTLKGAAKDVAGKLLEGLKCKITGCKP	31	3	3188.88	1.0000
**RaCa-3**	GLWETIKTTGKSIALNLLDKIKCKIAGGCPP	31	3	3269.95	1.0000
Ranatuerin-2C*	GVFLDTLKGLAGKMLESLKCKIAGCKP	27	3	2821.49	0.9999
**RaCa-4**	FLTFPGMTFGKLLGK	15	2	1657.05	0.9997
**RaCa-5**	GLLDIIKDTGKTTGILMDTLKCQMTGRCPPSS	32	1	3395.02	0.9996
**RaCa-6**	ATAWRIPPPGMQPIIPIRIRPLCGKQ	26	4	2910.58	0.9994
**RaCa-7**	FFPRVLPLANKFLPTIYCALPKSVGN	26	3	2906.52	0.9985
**RaCa-8**	FPAIICKVSKNC	12	2	1322.65	0.9961
**RaCa-9**	FYFPVSRKFGGK	12	3	1432.69	0.9412
**RaCa-10**	ALVAKIQKFPVFNTLKLCKLELEII	25	2	2872.59	0.6063
**RaCa-11**	SNRDFFKVNIFRLCG	15	2	1816.11	0.6058

*Previously reported amphibian peptide sequences [34, 38, 39]

^+^Previously reported as a full-length AMP precursor sequence. Uniprot ID: C5IB07

^a^Net charge at pH = 7

### Antimicrobial susceptibility testing (AST)

A panel composed of six bacteria was selected to test candidate AMP sequences identified using AMPlify: *Staphylococcus aureus* ATCC 6538P, *Streptococcus pyogenes* (unknown strain; hospital isolate), *Pseudomonas aeruginosa* ATCC 10148, *Escherichia coli* ATCC 9723H and ATCC 29522, and an MDR carbapenemase-producing New-Delhi metallobetalactamase (CPO-NDM) *Escherichia coli* clinical isolate. *E. coli* ATCC 29522 was used as a wild-type drug susceptible control strain. Results from AST are presented in Table 3. Supplementary Table S4 provides additional data with results shown in μg/mL.

**Table 3:** Minimum inhibitory concentrations (MIC) and minimum bactericidal concentrations (MBC) of selected AMP candidates following antimicrobial susceptibility testing (AST) in vitro. Candidate antimicrobial peptides were synthesized and purchased from Genscript. AST, and MIC/MBC determination was performed as outlined by the Clinical and Laboratory Standards Institute (CLSI) [40], with modification as recommended by Hancock [41]. Data is presented as the lowest effective peptide concentration range (μM) observed in three independent experiments. LL37, human cathelicidin and a peptide from Tp0751 from *Treponema pallidum* were used as the positive and negative control peptides [34], respectively

	***S. aureus***^a^ ATCC 6538P		***S. pyogenes***^b^		***P. aeruginosa***^a^ ATCC 10148		***E. coli***^a^ ATCC 9723H		***E. coli***^c^ ATCC 25922		MDR ***E. coli***^d^ (CPO-NDM)	
	Gram-positive		Gram-positive		Gram-negative		Gram-negative		Gram-negative		Gram-negative	
(μM)	MIC	MBC	MIC	MBC	MIC	MBC	MIC	MBC	MIC	MBC	MIC	MBC
**RaCa-1**	NI	NI	79	≥ 79	NI	NI	20 – 39	39 – 79	10 – 20	10 – 39	20 – 39	20 – 39
**RaCa-2**	1 – 2	1 – 2	25 – 49	25 – 49	25 – 49	49 – ≥99	3 – 6	3 – 6	2 – 6	2 – 6	2 – 6	2 – 6
**RaCa-3**	≥78	NI	39	39 – ≥ 78	20 – ≥78	39 – ≥78	5 – 10	5 – 10	2 – 5	2 – 5	5 – 10	5 – 20
**RaCa-4**	NI	NI	NI	NI	NI	NI	NI	NI	–	–	–	–
**RaCa-5**	NI	NI	NI	NI	NI	NI	NI	NI	NI	NI	NI	NI
**RaCa-6**	NI	NI	NI	NI	NI	NI	NI	NI	NI	NI	NI	NI
**RaCa-7**	≥ 88	NI	NI	NI	NI	NI	11 – 22	11 – 88	6 – 44	6 – 44	6 – 44	6 – 44
**RaCa-8**	NI	NI	NI	NI	NI	NI	NI	NI	NI	NI	NI	NI
**RaCa-9**	NI	NI	NI	NI	NI	NI	NI	NI	–	–	–	–
**RaCa-10**	NI	NI	NI	NI	NI	NI	NI	NI	NI	NI	NI	NI
**RaCa-11**	NI	NI	NI	NI	NI	NI	NI	NI	–	–	–	–
**LL37**	NI	NI	NI	NI	7 – ≥57	7 – ≥57	2 – 4	4 – 7	2 – 4	2 – 4	2 – 4	2 – 4
**Tp0751**	NI	NI	NI	NI	NI	NI	NI	NI	NI	NI	NI	NI

^a^Bacteria obtained and tested at the University of Victoria

^b^Unknown strain; hospital isolate

^c^ATCC quality control strain #25922 purchased from Cedarlane Laboratories (Burlington, Ontario, Canada)

^d^Clinical isolate obtained and tested at the British Columbia Centre for Disease Control

NI, no inhibition observed in vitro

‘—’ = not tested

Abbreviations: *Staphylococcus aureus*, *Streptococcus pyogenes*, *Pseudomonas aeruginosa*, *Escherichia coli*, *ATCC* American Type Culture Collection, *CPO* carbapenemase-producing organism, *MDR* multi-drug resistant, *NDM* New-Delhi Metallo-beta-lactamase

The 11 putative AMP sequences were selected for in vitro AST experiments, and four of them displayed antimicrobial activity against the targets tested: RaCa-1, RaCa-2, RaCa-3, and RaCa-7. RaCa-1 was antibacterial against all *E. coli* strains tested (MIC = 10–39 μM, MBC = 10–79 μM). RaCa-1 also showed minimal antimicrobial activity against *S. pyogenes* (MIC/MBC ≥ 79 μM) with no observed inhibition against the *S. aureus* and *P. aeruginosa* isolates. RaCa-2 and RaCa-3 inhibited all bacterial strains tested. RaCa-2 possessed the strongest antibacterial activity against *S. aureus* and *E. coli* isolates, preventing growth of both species of bacteria at concentrations of 1–2 μM and 2–6 μM, respectively. Specifically, this peptide was bactericidal against *E. coli* ATCC 9723H (MIC/MBC = 3–6 μM), with similar activity observed against *E. coli* ATCC 25922 and the MDR *E. coli* CPO-NDM isolates (MIC/MBC = 2–6 μM). RaCa-2 was also the only AMP tested to have robust bactericidal action against both *S. aureus* (MIC/MBC = 1–2 μM) and *S. pyogenes* (MIC/MBC = 25–49 μM). Comparably, RaCa-3 was considerably potent in vitro against *S. pyogenes* (MIC = 39 μM, MBC = 39–≥78 μM), *P. aeruginosa* (MIC = 20–≥78 μM, MBC = 39–≥78 μM), *E. coli* (MIC = 2–10 μM, MBC = 2–20 μM), and to a lesser extent *S. aureus* (MIC ≥78 μM, MBC = NI). RaCa-7 was active against all strains of *E. coli* (MIC = 6–44 μM, MBC = 6–88 μM), with minimal inhibition of *S. aureus* (MIC ≥88 μM, MBC = NI), and no activity against the other two species. Overall, the four novel AMP sequences displayed the strongest activity against the tested *E. coli* strains. RaCa-2 and RaCa-3 each had potent antibacterial action against the MDR *E. coli* (CPO-NDM) inhibiting bacterial growth at ≤10 μM. Of particular note, there was little or no observed shift in MIC and MBC values when comparing the CPO-NDM *E. coli* isolate to the ATCC 25922 wild-type control strain.

The positive control peptide LL37 [34] displayed potent antimicrobial activity against all strains of *E. coli* (MIC = 2–4 μM, MBC = 2–7 μM) and *P. aeruginosa* (MIC = 7–≥57 μM, MBC = 7–≥57 μM). However, this peptide had no activity against the tested strains of *S. aureus* and *S. pyogenes*, respectively. The negative control peptide, Tp0751, a non-functional truncated section of a *Treponema pallidum* protein with similar characteristics to AMPs [42], was inactive against all organisms.

## Discussion

Here we present AMPlify, a robust attentive deep learning model for AMP prediction, and demonstrate its utility in identifying novel AMPs with broad antimicrobial activities. It implements ensemble learning by partitioning its training set – a novel approach – and outperforms existing machine learning methods, including a leading deep learning based model. The two attention mechanisms in AMPlify are inspired by how humans perceive natural language, paying closer attention to regions or words of interest in a sentence. We have observed that single sub-models of AMPlify were able to outperform the state-of-the-art methods without ensemble learning, and we were able to trace the source of this favourable performance to the inclusion of attention layers.

Although machine learning methods in general, and AMPlify in particular, perform well in predicting AMPs, their performance can be limited by a paucity of detailed AMP sequence data available for training. First, the models do not usually consider the potential target microorganisms for the predicted AMPs. Although some methods report success at that level of granularity using public data [15, 16], incomplete and incorrect annotations in AMP databases are confounding. Second, the models cannot distinguish whether an AMP acts directly on biological membranes and/or by modulating the host immunity, since there is no consistently available data on these features. AMPs acting only in the latter mode require separate assays and might differ in activity within different species. Third, the size of the training data is still small relative to the data typically employed in most deep learning applications. Specially, having more similar sequences with different antimicrobial activities (i.e. non-AMPs that are similar to known AMPs) in the training set might help the model to be more sensitive to small changes in the sequences for prediction. However, availability of such information is limited. As a result, all the publicly available AMP prediction tools face difficulty in differentiating between AMPs and non-AMPs that are highly similar in their sequences (Supplementary Note S3, Supplementary Table S5). We expect this limitation to be gradually alleviated as more AMPs are discovered and more AMP mutation and truncation studies are done, inspired by tools like AMPlify. Although the size of the training data is unlikely to ever match what is available in natural language processing, image classification, and social network analysis domains, to name a few, AMP prediction tools can still find practical applications as demonstrated here.

Using AMPlify, four novel AMPs were identified with proven activity against a variety of bacterial isolates. Promisingly, two of the four presented AMPs demonstrate potent antibacterial activity against the MDR *E. coli* tested, and there was little or no observed shift in MIC when comparing the MDR and drug-susceptible strains. This suggests that the mechanism-of-action of these AMPs is unlike those used by conventional antibiotics. Thus, AMPs, such as those presented in the current study, have the potential to be used in future drug and clinical development studies as peptide-based substitutes to classical antibiotics. Although several candidates identified using this pipeline did not show any in vitro activity against the bacteria tested, we speculate that they still may possess activity against other bacterial species or other microorganisms (e.g. fungi, virus), or may demonstrate activity in vivo via host immune response modulation. Further, the structures of these sequences are highly dynamic and susceptible to change in response to the surrounding microenvironment, as is frequently the case with amphipathic alpha helices. These AMPs may act as monomers or form multimeric complexes, with their secondary structure flexibly changing in response to interaction with membranes or free divalent cations [43]. Further studies are required to interrogate AMP mechanisms as these phenomena are not readily observed using classical in vitro methods.

Of course, the utility of tools like AMPlify is not limited to discovering AMPs from the bullfrog genome; they can be generically applied to any input sequence. As such, they have the potential to play a role in de novo AMP design or enhancement. In conclusion, with their various use cases, we foresee tools like AMPlify as being instrumental in expanding the current arsenal of antimicrobial agents effective against WHO priority pathogens.

## Conclusions

This study introduces a novel attentive deep learning model, AMPlify, for AMP prediction, and has identified four novel AMPs from the bullfrog genome with promising antibacterial activity against an MDR WHO priority pathogen. We illustrate the value of attention mechanisms and a novel ensemble approach in mining genome resources for novel AMPs, comparing the performance of AMPlify to the state-of-the art machine learning models. AMPlify is released as an open source tool (https://github.com/bcgsc/AMPlify) under the GPL-3.0 license.

## Methods

### Generation of the datasets

We used publicly available AMP sequences to train and test AMP predictors. In order to build a non-redundant AMP dataset, we first downloaded all available sequences from two manually curated databases: Antimicrobial Peptide Database [44] (APD3, http://aps.unmc.edu/AP) and Database of Anuran Defense Peptides [39] (DADP, http://split4.pmfst.hr/dadp). Since APD3 is being frequently updated, we used a static version that was scraped from the website on March 20, 2019 comprising 3061 sequences. Version 1.6 of DADP contains 1923 distinct mature AMPs. We concatenated these two sets and removed duplicate sequences, producing a non-redundant (positive) set of 4173 distinct, mature AMP sequences, all 200 amino acid residues in length or shorter. AMPs that are highly similar to each other at the sequence level were kept as separate entries, since small changes in amino acid compositions may lead to large changes in AMP activity [45]. Also, it is important to maintain as big a dataset as possible for better training of a deep learning model [17].

Training and testing binary classification models require a negative set, a collection of peptides known not to have any antimicrobial activity. Since there are no sequence catalogs for peptides devoid of antimicrobial activity, studies in the field typically select their non-AMP sequences from UniProt [46] (https://www.uniprot.org). This may involve excluding several simple keywords (e.g. antimicrobial, antibiotic) to filter out potential AMPs [14, 15], or additionally removing all secretory proteins [26] as AMPs are characteristically secreted peptides [47]. The former proposition is not sufficiently rigorous, because AMP annotation is not consistent and varies between sources. While keyword filtering may leave in the set some differently annotated AMPs, filtering of secretory proteins creates a learning gap for the model regarding such proteins without antimicrobial activities. Thus, it is important to balance these two strategies when selecting non-AMP sequences.

We designed a rigorous selection strategy for our non-AMP sequences (Supplementary Fig. S3), using sequences from the UniProtKB/Swiss-Prot database [46] (2019_02 release), which only contains manually annotated and reviewed records from the UniProt database. First, we downloaded sequences that are 200 amino acid residues or shorter in length (matching the maximum peptide length in the AMP set), excluding those with annotations containing any of the 16 following keywords related to antimicrobial activities: {antimicrobial, antibiotic, antibacterial, antiviral, antifungal, antimalarial, antiparasitic, anti-protist, anticancer, defense, defensin, cathelicidin, histatin, bacteriocin, microbicidal, fungicide}. Second, duplicates and sequences with residues other than the 20 standard amino acids were removed. Third, a set of potential AMP sequences annotated with any of the 16 selected keywords were downloaded and compared with our candidate negative set. We noted instances where a sequence with multiple functions was annotated separately in multiple records within the database, and removed sequences in common between candidate non-AMPs and potential AMPs. The candidate non-AMP sequences were also checked against the positive set to remove AMP sequences that lack the annotation in UniProtKB/Swiss-Prot. Finally, 4173 sequences were sampled from the remaining set of 128,445 non-AMPs, matching the number and length distribution of sequences in the positive set. An exception to the length distribution matching occurred when the length of a particular AMP sequence did not have a perfect match in the set of non-AMP sequences. In these instances, we chose the non-AMP sequence with the closest length. The matched length distributions were selected so that the model did not learn to distinguish classes based on sequence lengths.

The positive and negative sets were both split 80%/20% (3338/835) into training and test sets, respectively. We note that AMP sequences in our test partition have no overlap with the training sets of iAMPpred and iAMP-2L, but do share 196 sequences with the training set of AMP Scanner Vr.2.

### Model implementation

AMPlify is implemented in Python 3.6.7, using Keras library 2.2.4 [48] with Tensorflow 1.12.0 [49] as the backend. The raw output of the model can be interpreted as a probability score, thus sequences with scores > 0.5 are considered as AMPs and those ≤0.5 as non-AMPs. We used binary cross-entropy as the loss function, and the Adam algorithm [50] for optimizing weights. Dropout technique [51] was applied during training to prevent the model from over-fitting. The original positive and negative training sets were both split into sets of {667, 667, 668, 668, 668} sequences, and five training and validation set pairs were constructed by leaving one set out for validation to build five single sub-models. To optimize computational time and avoid overfitting, we applied early stopping during the training of each single sub-model. The validation accuracy was monitored at each training epoch, and the training process was stopped if there appeared to be no improvement for the next 50 epochs. The model weights from the epoch with the best validation accuracy were selected as the optimal weights. The output probabilities from the five single sub-models were averaged to yield an ensemble model.

Reflecting the composition of the sequences in the positive and negative sets, AMPlify only considers sequence lengths of 200 or shorter containing the 20 standard amino acid residues.

### Hyperparameter tuning and model architecture

In deep neural networks, the optimal hyperparameters, unlike model weights, cannot be learned directly from the training process, but they do play an important role in model performance. Thus, various combinations of hyperparameters must be compared in order to select the best set. Here we applied stratified 5-fold cross-validation on the entire training set to tune the model and find the best set of hyperparameters for the model architecture, as well as for training settings, including dropout rates and optimizer settings. For a fair comparison, we kept the same splits of sequences within cross-validation across all hyperparameter combinations. During hyperparameter tuning, we monitored the average performance on the validation partitions of cross-validation. Note that these validation partitions within cross-validation are different from the validation sets for early stopping, while the latter being additionally derived from the training partitions during the cross-validation process. The set of hyperparameters with the highest average cross-validation accuracy was chosen to train the final prediction model.

The AMPlify architecture includes three main components: 1) a bidirectional long short-term memory (Bi-LSTM) layer, 2) a multi-head scaled dot-product attention (MHSDPA) layer, and 3) a context attention (CA) layer (Fig. 1). To convert the original peptides into a mathematically processable format, each sequence is represented by a series of one-hot encoded vectors over an alphabet of 20 amino acids, yielding **x**_**1**_, **x**_**2**_, …, **x**_**L**_, where *L* is the length of the sequence and each **x**_**t**_ is a 20-dimensional vector of zeros and ones with ‖**x**_**t**_‖_**1**_ = 1 (*t* = 1, 2, …, *L*). The Bi-LSTM layer takes those one-hot encoded vectors as input and encodes positional information for each residue from both forward and backward directions, and the output vector for each residue is represented as a concatenation of the vectors from both directions. The best tuned dimensionality for each direction of Bi-LSTM layer was 512, resulting in the entire Bi-LSTM layer to be *d*_*h*_ = 512 × 2 = 1024 dimensional. Outputs from all residue positions of the Bi-LSTM layer are returned as the input for the next layer. The best tuned dropout rate of 0.5 was applied to the input of the Bi-LSTM layer. Encoding from the Bi-LSTM layer for residues within a given sequence can be represented as a series of vectors ${\mathbf{h}}_{\mathbf{t}}\in {\mathbb{R}}^{d_h}$ (*t* = 1, 2, …, *L*), and the entire sequence can be represented as a matrix with all **h**_**t**_ s packed as

$$H={\left({\mathbf{h}}_{\mathbf{1}},{\mathbf{h}}_{\mathbf{2}},\dots, {\mathbf{h}}_{\mathbf{L}}\right)}^{\mathrm{T}}\in {\mathbb{R}}^{L\times {d}_h}.$$

Next, the MHSDPA layer searches for relations between different residues in *n* different representation subspaces [30] (i.e. different attention heads) to further encode the sequence, where *n* is a hyperparameter to be tuned. Each residue first gets an intermediate representation within each head by calculating a weighted average over transformed vectors of all residues derived from their Bi-LSTM representations. The results from each head are then concatenated and mapped back to the original dimensionality. We adapted Vaswani and co-workers’ approach [30] to calculate the attention weights and outputs for the MHSDPA layer. The implementation was adapted from the GitHub repository at https://github.com/CyberZHG/keras-multi-head, where rectified linear unit (ReLU) activation functions and bias terms were added to all linear transformations.

In further detail, to obtain attention weights for different residues of a sequence within a head *i*, we calculate a set of queries ${Q}^i\in {\mathbb{R}}^{L\times {d}_k}$, keys ${K}^i\in {\mathbb{R}}^{L\times {d}_k}$, and values ${V}^i\in {\mathbb{R}}^{L\times {d}_v}$ by transforming *H* as follows:

$${Q}^i=\mathrm{ReLU}\left(H{W}^{Q^i}+{B}^{Q^i}\right)$$

$${K}^i=\mathrm{ReLU}\left(H{W}^{K^i}+{B}^{K^i}\right)$$

$${V}^i=\mathrm{ReLU}\left(H{W}^{V^i}+{B}^{V^i}\right)$$

where ${W}^{Q^i},{W}^{K^i}\in {\mathbb{R}}^{d_h\times {d}_k}$ and ${W}^{V^i}\in {\mathbb{R}}^{d_h\times {d}_v}$ are weight matrices, and ${B}^{Q^i}={\left({\mathbf{b}}^{{\mathbf{Q}}^{\mathbf{i}}},{\mathbf{b}}^{{\mathbf{Q}}^{\mathbf{i}}},\dots, {\mathbf{b}}^{{\mathbf{Q}}^{\mathbf{i}}}\right)}^{\mathrm{T}}\in {\mathbb{R}}^{L\times {d}_k}$, ${B}^{K^i}={\left({\mathbf{b}}^{{\mathbf{K}}^{\mathbf{i}}},{\mathbf{b}}^{{\mathbf{K}}^{\mathbf{i}}},\dots, {\mathbf{b}}^{{\mathbf{K}}^{\mathbf{i}}}\right)}^{\mathrm{T}}\in {\mathbb{R}}^{L\times {d}_k}$ and ${B}^{V^i}={\left({\mathbf{b}}^{{\mathbf{V}}^{\mathbf{i}}},{\mathbf{b}}^{{\mathbf{V}}^{\mathbf{i}}},\dots, {\mathbf{b}}^{{\mathbf{V}}^{\mathbf{i}}}\right)}^{\mathrm{T}}\in {\mathbb{R}}^{L\times {d}_v}$ are bias matrices. We set transformation dimensions as *nd*_*k*_ = *nd*_*v*_ = *d*_*h*_ following what has been previously proposed [30]. A square matrix *A*^*i*^ ∈ *ℝ*^*L* × *L*^, which contains weight vectors to calculate intermediate representations of all residues within head *i*, is computed as:

$${A}^i={\mathrm{softmax}}_{\mathrm{row}}\left(\frac{Q^i{K^i}^{\mathrm{T}}}{\sqrt{d_k}}\right)$$

where dot-product of queries and keys are scaled by a factor $\frac{1}{\sqrt{d_k}}$, and the softmax function is applied to each row of the matrix for normalization. The intermediate representation of the sequence within head *i* is then computed by:

$${Z}^i={\left({\mathbf{z}}_{\mathbf{1}}^{\mathbf{i}},{\mathbf{z}}_{\mathbf{2}}^{\mathbf{i}},\dots, {\mathbf{z}}_{\mathbf{L}}^{\mathbf{i}}\right)}^{\mathrm{T}}={A}^i{V}^i\in {\mathbb{R}}^{L\times {d}_v}$$

where each single vector ${\mathbf{z}}_{\mathbf{t}}^{\mathbf{i}}\in {\mathbb{R}}^{{\mathrm{d}}_v}$ (*t* = 1, 2, …, *L*) denotes the intermediate representation of each residue of the sequence with dimensionality *d*_*v*_. The concatenated matrix $Z=\left({Z}_{L\times {d}_v}^1,{Z}_{L\times {d}_v}^2,\dots, {Z}_{L\times {d}_v}^n\right)\in {\mathbb{R}}^{L\times {nd}_v}$ is further transformed to get the final output of the current layer as follows:

$$M={\left({\mathbf{m}}_{\mathbf{1}},{\mathbf{m}}_{\mathbf{2}},\dots, {\mathbf{m}}_{\mathbf{L}}\right)}^{\mathrm{T}}=\mathrm{ReLU}\left[Z{W}^O+{B}^O\right]\in {\mathbb{R}}^{L\times {d}_h}$$

where ${W}^O\in {\mathbb{R}}^{nd_v\times {d}_h}$ is a weight matrix and ${B}^O={\left({\mathbf{b}}^{\mathbf{O}},{\mathbf{b}}^{\mathbf{O}},\dots, {\mathbf{b}}^{\mathbf{O}}\right)}^{\mathrm{T}}\in {\mathbb{R}}^{L\times {d}_h}$ is a bias matrix. Each vector ${\mathbf{m}}_{\mathbf{t}}\in {\mathbb{R}}^{{\mathrm{d}}_h}$ (*t* = 1, 2, …, *L*) denotes the new representation of the corresponding residue of the sequence with dimensionality *d*_*h*_. The best head number tuned for this layer was *n* = 32, with *d*_*k*_ = *d*_*v*_ = 32.

Finally, the CA layer gathers information from the MHSDPA layer by reducing *L* encoded vectors into a single weighted average summary vector **s**. We followed Yang and co-workers’ approach [31] to perform this operation, and adapted the implementation from the GitHub repository at https://github.com/lzfelix/keras_attention. The weight vector **α** ∈ *ℝ*^*L*^ is calculated using

$$\boldsymbol{\upalpha} =\mathrm{softmax}\left(\left(\tanh \left( MW+B\right)\right)\mathbf{u}\right)$$

where $W\in {\mathbb{R}}^{d_h\times {d}_h}$ is a weight matrix, $B={\left(\mathbf{b},\mathbf{b},\dots, \mathbf{b}\right)}^{\mathrm{T}}\in {\mathbb{R}}^{L\times {d}_h}$ is a bias matrix, $\mathbf{u}\in {\mathbb{R}}^{d_h}$ is a context vector, and the softmax function provides weight normalization. The summary vector $\mathbf{s}\in {\mathbb{R}}^{d_h}$ is then computed as:

$$\mathbf{s}={M}^{\mathrm{T}}\boldsymbol{\upalpha} =\sum_{t=1}^L{\alpha}_t{\mathbf{m}}_{\mathbf{t}}$$

where *α*_*t*_ denotes each component in the weight vector. Vector **s** summarizes information of the entire sequence into a single vector, and it is passed through the output layer of a single neuron with a sigmoid activation function for classification. The best tuned dropout rate of 0.2 was applied to the input of the CA layer during training.

In addition to the hyperparameters of the model architecture, the hyperparameters of the optimizer were optimized through cross-validation. A batch size of 32 and a default learning rate of 0.001 were found to be the best for the AMP prediction task.

### Model evaluation

The performance of AMPlify was evaluated using five metrics: accuracy, sensitivity, specificity, F1 score and area under the receiver operating characteristic curve (AUROC).

The architecture of AMPlify was compared with its simpler variations with fewer hidden layers using stratified 5-fold cross-validation on the training set to measure the value added by each layer as the architecture grew more complex. The final version of AMPlify trained on the entire training set, as well as its five single sub-models, were compared with three other tools: iAMP-2L [15], iAMPpred [16] and AMP Scanner Vr.2 [26], on the test set we built. All comparators were evaluated with their original models online.

In addition, as the only comparator with methods for re-training, AMP Scanner Vr.2 was cross-validated and re-trained on our training set for a fairer comparison. We note that, since our dataset is slightly different from those used by other methods, the number of epochs required to get a deep learning model well trained on different datasets might differ. Keeping all other hyperparameters the same as the original model, we cross-validated and re-trained AMP Scanner Vr.2 with two different stopping settings: using the optimal fixed number of epochs as reported [26], and using early stopping.

### AMP discovery pipeline

A primarily homology-based approach was used to supply novel candidate AMP sequences to AMPlify for further evaluation. The pipeline and its results are summarized in Supplementary Fig. S4 and are detailed below.

Sequences matching the search phrase “((antimicrobial) AND precursor) AND amphibian” were downloaded from the NCBI Nucleotide database on January 4th, 2016 and aligned to the draft bullfrog genome [33] (version 3) using GMAP [52] (version 20170424) with the following parameters: -A --max-intronlength-ends = 200000 -O -n20 --nofails.

To refine the putative AMP loci, the gene prediction pipeline MAKER2 [53] (version 2.31.8 running under PERL version 5.24.0 with augustus [54] version 3.2.1, exonerate [55] version 2.2.0, genemark [56] version 2.3c, and snap [57] version 2006-07-28) was applied to the 231 genomic scaffolds with alignment hits from GMAP using default settings. The MAKER2 pipeline can use orthogonal evidence from related protein or transcript sequences when available to generate a list of high confidence genes. Protein evidence consisted of three sets of sequences: sequences matching the search phrase “((antimicrobial) AND precursor) AND amphibian” from the NCBI protein database that were downloaded on December 31st, 2015; experimentally validated non-synthetic amphibian antibacterial peptide sequences downloaded from CAMP [13] on March 4th, 2016; and sequences from APD3 [44] downloaded on September 29th, 2017. For transcript evidence, the set of cDNA sequences supplied to GMAP above was supplemented with selected bullfrog transcript sequences from the Bullfrog Annotation Reference for the Transcriptome [33] (BART). Blastn [58] (version 2.31.1) was used to align the initial cDNA sequences from NCBI to BART, and BART sequences with an alignment of greater than 90% identity and 100% coverage were selected. A custom repeat element library was constructed from predicted repeats previously identified in the bullfrog genome [33] and supplied to MAKER2 for use by RepeatMasker [59]. The annotation pipeline was run with the snap hidden Markov model that produced the version 2 bullfrog gene predictions [33].

The MAKER2 gene predictions were filtered in two stages. First, sequences containing the highly conserved lysine-arginine enzymatic cleavage motif were selected and the sequence of the putative mature peptide, produced via in silico cleavage at the C-terminal side of the cleavage motif, was extracted. Second, only putative mature sequences of 200 amino acid residues or less were included. Sequences with non-standard amino acid residues were excluded. The resulting peptide sequences from these filters were fed into AMPlify for prediction. From the predicted putative AMPs, only short cationic sequences with lengths between five and 35 amino acid residues were chosen for synthesis and validation in vitro. We prioritized short cationic sequences as shorter sequences are more structurally stable in various environments (e.g. in vitro and in vivo) [60] lending to easier therapeutic applicability.

### Antimicrobial susceptibility testing (AST)

From the novel candidate AMP sequences predicted by AMPlify, 11 were selected for validation in vitro. Minimum inhibitory concentrations (MIC) and minimum bactericidal concentrations (MBC) were obtained using the AST procedures outlined by the Clinical and Laboratory Standards Institute (CLSI) [40], with the recommended adaptations for testing cationic AMPs described by Hancock [41].

#### Bacterial isolates

A panel of two Gram-positive and four Gram-negative bacterial isolates was generated to test predicted AMPs. *Staphylococcus aureus* ATCC 6538P, *Streptococcus pyogenes* (hospital isolate, unknown strain), *Pseudomonas aeruginosa* ATCC 10148, and *Escherichia coli* ATCC 9723H were obtained and tested at the University of Victoria. Additionally, a multi-drug resistant (MDR), carbapenemase-producing New-Delhi metallobetalactamase (CPO-NDM) clinical isolate of *Escherichia coli* was obtained from the BC Centre for Disease Control. *E. coli* ATCC 29522 was purchased from Cedarlane Laboratories (Burlington, Ontario, Canada) for comparison of AMP activity between a wild type, drug-susceptible control and the MDR strain. The latter two strains were tested at the BC Centre for Disease Control using identical AST procedures.

#### Determination of MIC

Bacteria were streaked onto nonselective nutrient agar from frozen stocks and incubated for 18–24 h at 37 °C. To prepare a standardized bacterial inoculum, isolated colonies were suspended in Mueller-Hinton Broth (MHB) and adjusted to an optical density of 0.08–0.1 at 600 nm, equivalent to a 0.5 McFarland standard and representing approximately 1–2 × 10^8^ CFU/mL (CFU: colony forming units). The inoculum was diluted 1/250 in MHB to the target concentration of (5 ± 3) × 10^5^ CFU/mL. Total viability counts from the final inoculum were examined to confirm the target bacterial density was obtained.

Selected candidate AMPs were purchased from Genscript (Piscataway, NJ), where they were synthesized using the vendor’s Flexpeptide platform. Lyophilized peptides were suspended in sterile ultrapure water or filter-sterilized 0.2% acetic acid as recommended by solubility testing reports provided with the GenScript synthesis. AMPs were diluted from 2560 to 5 μg/mL by a two-fold serial dilution in a 96-well polypropylene microtitre plate before 100 μl of the standardized bacterial inoculum of (5 ± 3) × 10^5^ CFU/mL was added to each well. This generated a final test range of 256 to 0.5 μg/mL. MIC values were reported as the peptide concentration that produced no visible bacterial growth after a 16–24 h incubation at 37 °C.

#### Determination of MBC

For each AMP dilution series, the contents of the MIC well and the two adjacent wells containing two- and four-fold MIC were plated onto nonselective nutrient agar and incubated for 24 h at 37 °C. The concentration which killed 99.9% of the initial inoculum was determined to be the MBC.

### Supplementary Information

**Additional file 1: Supplementary Note S1**: Performance comparison of AMP Scanner Vr.2 and AMPlify re-trained on the AMP Scanner Vr.2 dataset. **Supplementary Note S2**: Performance comparison of different AMP prediction tools based on the test sequence similarities to their corresponding training sets. **Supplementary Note S3**: Comparison of different AMP prediction tools tested on similar sequences with different labels. **Supplementary Figure S1**: Learning curve comparison (on the validation sets for early stopping) of single sub-models of AMPlify trained on two different datasets. (a) Single sub-models of AMPlify trained on our own training set; (b) Single sub-models of AMPlify trained on the AMP Scanner Vr.2 “Train+Tune” partitions. Square markers denote the best epochs chosen by early stopping, and the x-axes have been set in the same range in order for a clearer comparison. **Supplementary Figure S2**: UpSet plot of the 101 candidate mature sequences with regard to the three filters. This plot visualizes the results obtained by applying different combinations of filters to the 101 candidate mature sequences. **Supplementary Figure S3**: Workflow of selecting the non-AMP sequences from the UniProtKB/Swiss-Prot database. **Supplementary Figure S4**: Workflow of the AMP discovery pipeline. The process describes how 75 putative AMPs were identified from the bullfrog genome. Invalid sequences denote those not suitable for AMPlify prediction, with lengths outside the range 2 to 200 amino acids or with non-standard amino acids. **Supplementary Table S1**: Stratified 5-fold cross-validation results of different architectures on the training set. The top section compares the architecture of AMPlify, with and without ensemble learning, with its simpler variations. The second section shows the architecture of AMP Scanner Vr.2 cross-validated on our training set. Values of accuracy (acc), sensitivity (sens), specificity (spec), F1 score (F1) and area under the receiver operating characteristic curve (AUROC) are presented along with their standard deviations in percentage. **Supplementary Table S2**: Comparison between AMP Scanner Vr.2 and AMPlify cross-validated on all data provided by AMP Scanner Vr.2 (“Train+Tune+Test” partitions). This table shows the 10-fold cross-validation results of AMP Scanner Vr.2 and AMPlify on all data provided by AMP Scanner Vr.2. Values of accuracy (acc), sensitivity (sens), specificity (spec) and area under the receiver operating characteristic curve (AUROC) are presented along with their standard deviations in percentage. **Supplementary Table S3**: Performance comparison between AMP Scanner Vr.2 and AMPlify re-trained on the AMP Scanner Vr.2 “Train+Tune” partitions and tested on their “Test” partition. Since AMPlify applies early stopping and the exact size of training set for each single sub-model is smaller, the exact training size for each model is listed here in the second column. Values of accuracy (acc), sensitivity (sens), specificity (spec) and area under the receiver operating characteristic curve (AUROC) are presented in percentage. **Supplementary Table S4**: Minimum inhibitory concentrations (MIC) and minimum bactericidal concentrations (MBC) of selected AMP candidates following antimicrobial susceptibility testing (AST) in vitro. This is a supplementary table to Table 3. Candidate antimicrobial peptides were synthesized and purchased from Genscript. AST, and MIC/MBC determination was performed as outlined by the Clinical and Laboratory Standards Institute (CLSI), with modification as recommended by Hancock. Data is presented as the lowest effective peptide concentration range (μg/mL) observed in three independent experiments. LL37, human cathelicidin and a peptide from Tp0751 from *Treponema pallidum* were used as the positive and negative control peptides, respectively. **Supplementary Table S5**: Predictions of Gaegurin 5 (GGN5) and its analogues by different AMP prediction tools. Antimicrobial activity data of GGN5 and its analogues were taken from the work by Won and con-workers. The analogues were generated by truncating the parent peptide into shorter fragments and/or by amino acid substitutions. Prediction results of AMPlify, AMP Scanner Vr.2, and iAMPpred were listed for comparison.

## Data Availability

The source code for AMPlify and the trained models are available at https://github.com/bcgsc/AMPlify.

## Abbreviations

Acc: Accuracy
AMP: Antimicrobial peptide
APD: Antimicrobial Peptide Database
AST: Antimicrobial susceptibility testing
ATCC: American Type Culture Collection
AUROC: Area under the receiver operating characteristic curve
BART: Bullfrog Annotation Reference for the Transcriptome
Bi-LSTM: Bidirectional long short-term memory
CA: Context attention
CAMP: Collection of Antimicrobial Peptides
CLSI: Clinical and Laboratory Standards Institute
CNN: Convolutional neural network
CPO: Carbapenemase-producing organism
DADP: Database of Anuran Defense Peptides
LSTM: Long short-term memory
MBC: Minimum bactericidal concentration
MDR: Multi-drug resistant
MHB: Mueller-Hinton Broth
MHSDPA: Multi-head scaled dot-product attention
MIC: Minimum inhibitory concentration
MRSA: Methicillin-resistant *Staphylococcus aureus*
NDM: New-Delhi Metallo-beta-lactamase
PseAAC: Pseudo amino acid compositions
RNN: Recurrent neural network
ROC: Receiver operating characteristic
Sens: Sensitivity
Spec: Specificity
VRSA: Vancomycin-resistant *Staphylococcus aureus*
WHO: World Health Organization
