# Supplementary material for: AMPlify: attentive deep learning model for discovery of novel antimicrobial peptides effective against WHO priority pathogens
Source: BMC Genomics. 2022 Jan 25;23:77. doi: 10.1186/s12864-022-08310-4 (PMC8788131; doi:10.1186/s12864-022-08310-4)
Supplement: Supplementary file 1 — Additional file 1: Supplementary Note S1: Performance comparison of AMP Scanner Vr.2 and AMPlify re-trained on the AMP Scanner Vr.2 dataset. Supplementary Note S2: Performance comparison of different AMP prediction tools based on the test sequence similarities to their corresponding training sets. Supplementary Note S3: Comparison of different AMP prediction tools tested on similar sequences with different labels. Supplementary Figure S1: Learning curve comparison (on the validation sets for early stopping) of single sub-models of AMPlify trained on two different datasets. (a) Single sub-models of AMPlify trained on our own training set; (b) Single sub-models of AMPlify trained on the AMP Scanner Vr.2 “Train+Tune” partitions. Square markers denote the best epochs chosen by early stopping, and the x-axes have been set in the same range in order for a clearer comparison. Supplementary Figure S2: UpSet plot of the 101 candidate mature sequences with regard to the three filters. This plot visualizes the results obtained by applying different combinations of filters to the 101 candidate mature sequences. Supplementary Figure S3: Workflow of selecting the non-AMP sequences from the UniProtKB/Swiss-Prot database. Supplementary Figure S4: Workflow of the AMP discovery pipeline. The process describes how 75 putative AMPs were identified from the bullfrog genome. Invalid sequences denote those not suitable for AMPlify prediction, with lengths outside the range 2 to 200 amino acids or with non-standard amino acids. Supplementary Table S1: Stratified 5-fold cross-validation results of different architectures on the training set. The top section compares the architecture of AMPlify, with and without ensemble learning, with its simpler variations. The second section shows the architecture of AMP Scanner Vr.2 cross-validated on our training set. Values of accuracy (acc), sensitivity (sens), specificity (spec), F1 score (F1) and area under the receiver operating characteristic curv [file 12864_2022_8310_MOESM1_ESM.pdf]

Supplementary Materials

AMPlify: attentive deep learning model for discovery of novel antimicrobial peptides effective against WHO priority pathogens

Chenkai Li[1, 2], Darcy Sutherland[1, 3, 4], S. Austin Hammond[1, †], Chen Yang[1, 2], Figali Taho[1, 2], Lauren Bergman[5], Simon Houston[5], René L. Warren[1], Titus Wong[4, 6], Linda M.N. Hoang[3, 4], Caroline E. Cameron[5, 7], Caren C. Helbing[5], and Inanc Birol[1, 3, 4, 8, *]

1. Canada's Michael Smith Genome Sciences Centre, BC Cancer Agency, Vancouver, BC, V5Z 4S6, Canada
2. Bioinformatics Graduate Program, University of British Columbia, Vancouver, BC, V6T 1Z4, Canada
3. Public Health Laboratory, British Columbia Centre for Disease Control, Vancouver, BC, V5Z 4R4, Canada
4. Department of Pathology and Laboratory Medicine, University of British Columbia, Vancouver, BC, V6T 1Z4, Canada
5. Department of Biochemistry and Microbiology, University of Victoria, Victoria, BC, V8P 5C3, Canada
6. Medical Microbiology Laboratory, Vancouver General Hospital, Vancouver, BC, V5Z 1M9, Canada
7. Division of Infectious Diseases, Department of Medicine, University of Washington, Seattle, WA, 98195, USA
8. Department of Medical Genetics, University of British Columbia, Vancouver, BC, V6H 3N1, Canada

† Current address: Next-Generation Sequencing Facility, University of Saskatchewan, Saskatoon, SK, S7N 5E5, Canada
* Correspondence: Inanc Birol (ibirol@bcgsc.ca)

Supplementary Notes

Supplementary Note S1: Performance comparison of AMP Scanner Vr.2 and AMPlify re-trained on the AMP Scanner Vr.2 dataset

In order to test whether the AMPlify architecture can still perform well on other datasets, we conducted an analysis on the AMP Scanner Vr.2 datasets [1] (named "Train", "Tune" and "Test" partitions) in two different ways. First, AMPlify was cross-validated on all data provided by AMP Scanner Vr.2 in the same way they did in their study [1]. Second, AMPlify was re-trained on the dataset ("Train+Tune" partitions) used in the original AMP Scanner Vr.2 publication for performance comparison on the "Test" partition [1]. The results for AMP Scanner Vr.2 were taken directly from Table 1 of Veltri and co-workers' work [1], with the best tuned hyperparameters. They did not report the F1 score, so this metric is not compared here.

Supplementary Table S2 shows the 10-fold cross-validation results of AMP Scanner Vr.2 and AMPlify on all data provided by AMP Scanner Vr.2. A single sub-model of AMPlify without ensemble learning still outperforms AMP Scanner Vr.2 in accuracy (92.15% vs. 91.51%), sensitivity (91.06% vs. 88.81%) and AUROC (97.15% vs. 96.58%), although its specificity is slightly lower, with a difference < 1%. After ensemble learning is adopted, AMPlify outperforms AMP Scanner Vr.2 in all four metrics by at least 1% and as much as 1.74%.

Supplementary Table S3 shows the performance comparison on the AMP Scanner Vr.2 test set between AMP Scanner Vr.2 and the re-trained version (on "Train+Tune" partitions) of AMPlify. AMPlify re-trained on their dataset still outperforms AMP Scanner Vr.2 in all four metrics of accuracy (92.35% vs. 91.01%), sensitivity (90.59% vs. 89.89%), specificity (94.10% vs. 92.13%) and AUROC (97.00% vs. 96.48%). However, single sub-models of AMPlify re-trained on their dataset does not show substantial advantage in overall accuracy over AMP Scanner Vr.2. First, the re-trained version of AMPlify here is trained on fewer data than AMP Scanner Vr.2 due to the early stopping technique used. Second, the validation sets we set aside for early stopping here are too small compared with those used in our own training process, as the entire set for training here is only 1/3 of ours in size. Note that the 10-fold cross-validation does not suffer from such problems because it is done on all data, with both the training and the early stopping monitoring validation sets of single sub-model within each fold larger. After a train-validation split for early stopping, the new training and early stopping monitoring validation sets for test comparison comprised only 1704–1706 and 426–428 sequences respectively, while those for 10-fold cross-validation used 2560–2562 and 640–642 sequences respectively. Supplementary Fig. S1 shows a comparison between the learning curves of five single sub-models trained on our own training set and on their original dataset, respectively. Obviously, the validation accuracy fluctuates greatly when the models are trained on their dataset, which suggests that those validation sets are too small to represent the distribution of the entire dataset. Hence, single sub-models of AMPlify are not well trained on their dataset since it is difficult for early stopping to decide the best epochs in such cases. Thus, we conclude that their dataset is not sufficiently large to train and validate AMPlify.

In summary, AMPlify and its single sub-models can outperform the state-of-the-art method, AMP Scanner Vr.2, with respect to the 10-fold cross-validation analysis on their dataset. Further, AMPlify re-trained on their dataset is still able to outperform AMP Scanner Vr.2, and arguably the single sub-models of AMPlify were likely to gain better performance if the training set were large enough.

Supplementary Note S2: Performance comparison of different AMP prediction tools based on the test sequence similarities to their corresponding training sets

In order to evaluate how similarities to the training peptides in sequence level affect the model performance on the test sequences, subsets of our original AMP/non-AMP test sets were generated by choosing different similarity thresholds to the corresponding AMP/non-AMP training sets of the tools. The similarity between two sequences was calculated as $1 - \frac{d_{i,j}}{\max(l_i, l_j)}$, where $d_{i,j}$ is the edit distance and l_i, l_j are lengths of the sequences. The similarity of a sequence to a dataset of sequences was defined as the maximum of all similarity values calculated between that sequence and the sequences in the dataset. Note that different tools may have different test subsets under the same similarity threshold, since they were trained on different training sets. Each AMP/non-AMP test subset contained test sequences that shared similarities less than or equal to a given threshold to the corresponding AMP/non-AMP training set. Combining these sets, we have measured the F1 scores of the tools (Fig. 2). Here, AMPlify obtains the highest F1 scores despite the similarity threshold chosen, which reveals that AMPlify captures other high-level features besides the sequence similarity information.

Supplementary Note S3: Comparison of different AMP prediction tools tested on similar sequences with different labels

To evaluate the ability of different AMP prediction tools in differentiating similar sequences with different labels (AMP or non-AMP), we tested AMPlify and its comparators on a set of 32 sequences containing a known AMP Gaegurin 5 (GGN5) and its 31 analogues from the work by Won and co-workers [2]. The 31 analogues of GGN5 were generated by truncating the parent peptide into shorter fragments and/or by amino acid substitutions. Among the 32 sequences, 21 of them showed antimicrobial activity against at least one of the ten bacterial species tested, while the rest 11 did not show any activity [2].

AMPlify and other tools predict all those 32 peptides as AMPs (Supplementary Table 5). We note that the online server of iAMP-2L [3] was down at the time this analysis was done (Nov 7, 2021), so its results were not compared here. These results indicate that all the current AMP prediction tools have limitation in identifying AMPs and non-AMPs that are highly similar in their sequences. This is likely due to the lack of experimentally validated data for highly similar AMP and non-AMP sequences in the public database for model training. We expect the limitation to be gradually alleviated when more and more related studies for AMP mutation and truncation are done in the future.

Supplementary Figures

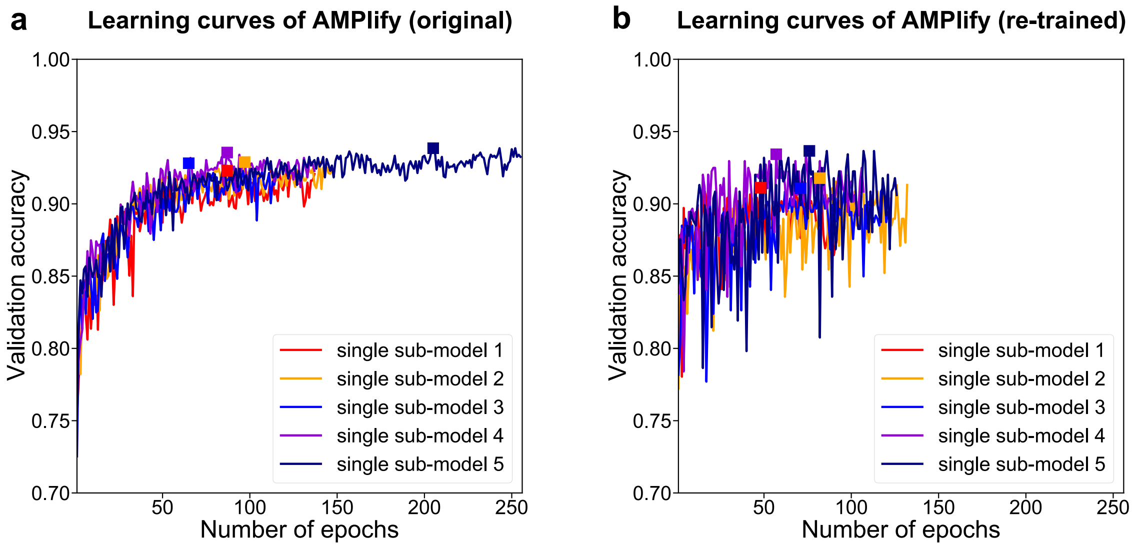

Supplementary Fig. S1: Learning curve comparison (on the validation sets for early stopping) of single sub-models of AMPlify trained on two different datasets. (a) Single sub-models of AMPlify trained on our own training set; **(b)** Single sub-models of AMPlify trained on the AMP Scanner Vr.2 "Train+Tune" partitions. Square markers denote the best epochs chosen by early stopping, and the x-axes have been set in the same range in order for a clearer comparison.

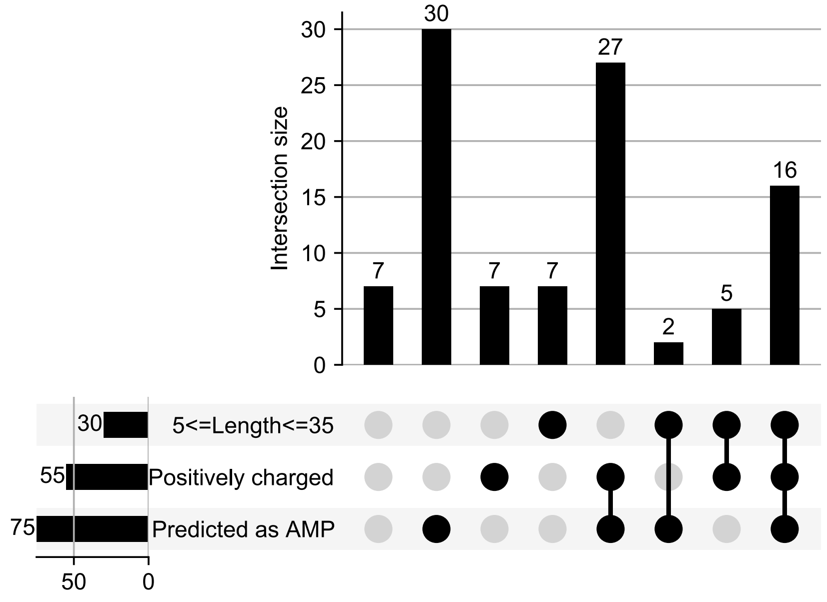

Supplementary Fig. S2: UpSet plot of the 101 candidate mature sequences with regard to the three filters. This plot visualizes the results obtained by applying different combinations of filters to the 101 candidate mature sequences.

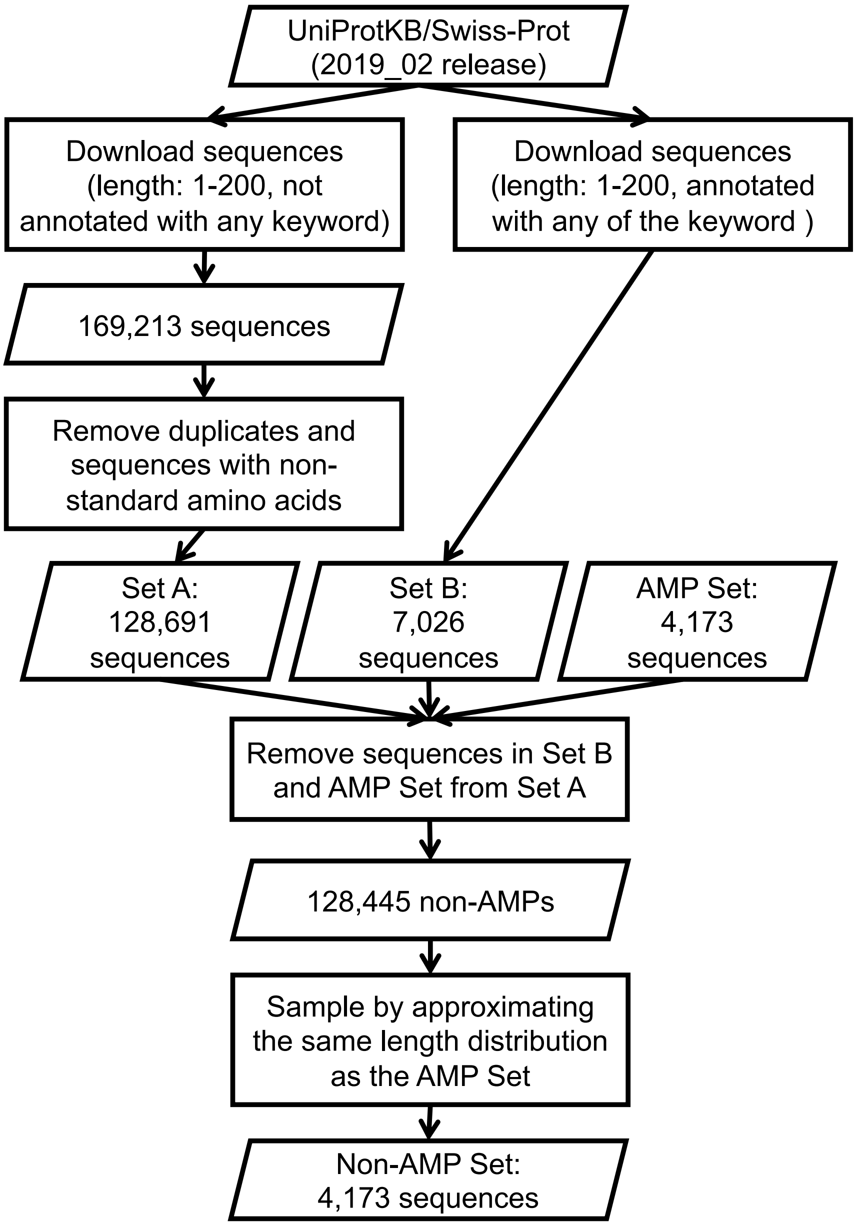

Supplementary Fig. S3: Workflow of selecting the non-AMP sequences from the UniProtKB/Swiss-Prot database.

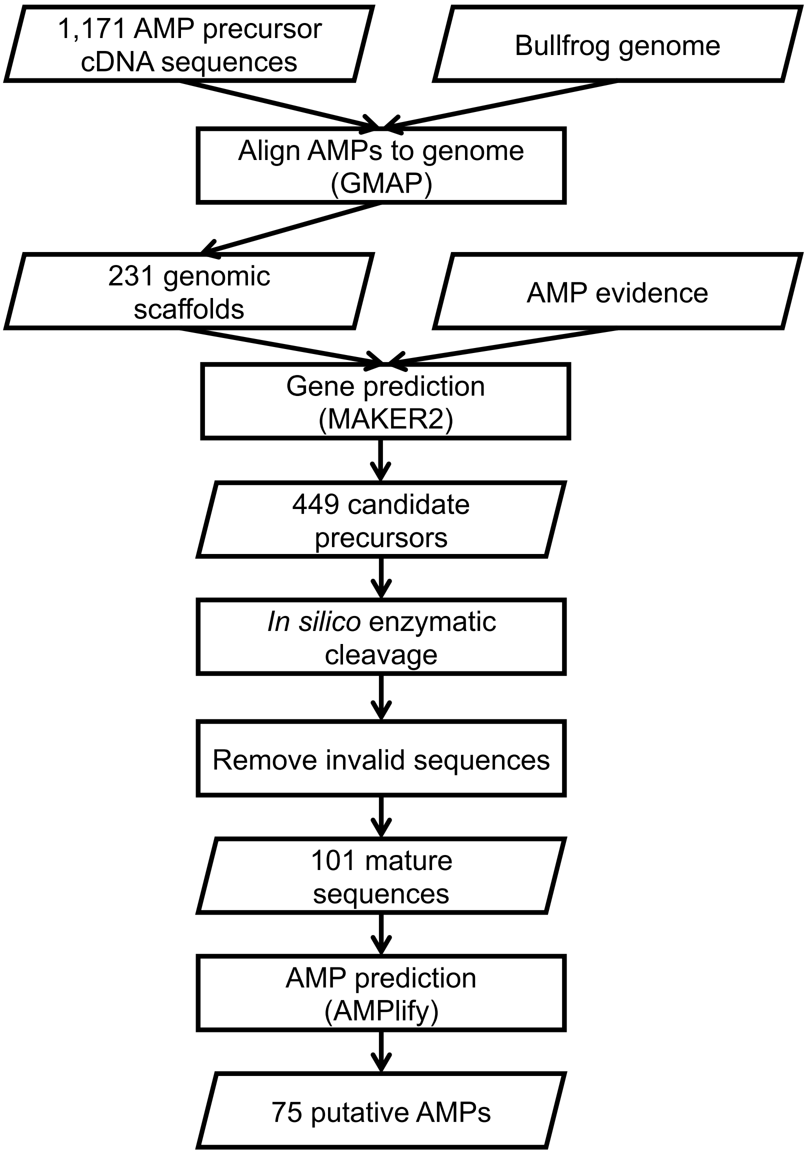

Supplementary Fig. S4: Workflow of the AMP discovery pipeline. The process describes how 75 putative AMPs were identified from the bullfrog genome. Invalid sequences denote those not suitable for AMPlify prediction, with lengths outside the range 2 to 200 amino acids or with non-standard amino acids.

Supplementary Tables

Supplementary Table S1: Stratified 5-fold cross-validation results of different architectures on the training set. The top section compares the architecture of AMPlify, with and without ensemble learning, with its simpler variations. The second section shows the architecture of AMP Scanner Vr.2 cross-validated on our training set. Values of accuracy (acc), sensitivity (sens), specificity (spec), F1 score (F1) and area under the receiver operating characteristic curve (AUROC) are presented along with their standard deviations in percentage.

Model Architecture	Acc	Sens	Spec	F1	AUROC
Bi-LSTM	89.04±1.02	89.10±1.14	88.98±1.80	89.05±0.98	95.82±0.56
Bi-LSTM + CA	90.13±1.02	90.05±0.94	90.20±1.91	90.13±0.95	96.14±0.37
Bi-LSTM + MHSDPA + CA[a]	91.70±0.66	91.40±0.71	92.00±1.51	91.68±0.61	96.92±0.30
**AMPlify**: Bi-LSTM + MHSDPA + CA (with ensemble)	**92.79±0.77**	**92.12±1.28**	**93.47±0.53**	**92.74±0.80**	**97.44±0.53**
**AMP Scanner Vr.2:** Embedding + Conv + Max Pooling + LSTM					
- [10 epochs[b]]	90.55±1.43	90.14±1.62	90.95±4.08	90.54±1.17	96.40±0.58
- [early stopped[c]]	90.52±0.76	89.45±1.10	91.58±1.48	90.42±0.74	96.35±0.43

[a]Trained without ensemble learning (i.e. single sub-model).
[b]The best hyperparameter as stated in the paper.
[c]Optimal numbers of training epochs determined by early stopping range from 17 to 71.

Supplementary Table S2: Comparison between AMP Scanner Vr.2 and AMPlify cross-validated on all data provided by AMP Scanner Vr.2 ("Train+Tune+Test" partitions). This table shows the 10-fold cross-validation results of AMP Scanner Vr.2 and AMPlify on all data provided by AMP Scanner Vr.2. Values of accuracy (acc), sensitivity (sens), specificity (spec) and area under the receiver operating characteristic curve (AUROC) are presented along with their standard deviations in percentage.

Model	Acc	Sens	Spec	AUROC
AMP Scanner Vr.2	91.51±0.89	88.81±3.53	94.21±2.68	96.58±0.66
AMPlify (re-trained, single sub-model)	92.15±0.86	**91.06±1.76**	93.25±2.51	97.15±0.58
AMPlify (re-trained, ensemble)	**93.22±0.71**	90.55±1.62	**95.89±1.56**	**97.61±0.46**

Supplementary Table S3: Performance comparison between AMP Scanner Vr.2 and AMPlify re-trained on the AMP Scanner Vr.2 "Train+Tune" partitions and tested on their "Test" partition. Since AMPlify applies early stopping and the exact size of training set for each single sub-model is smaller, the exact training size for each model is listed here in the second column. Values of accuracy (acc), sensitivity (sens), specificity (spec) and area under the receiver operating characteristic curve (AUROC) are presented in percentage.

Model	Training set size	Acc	Sens	Spec	AUROC
AMP Scanner Vr.2	2132	91.01	89.89	92.13	96.48
AMPlify (re-trained)	2132	**92.35**	90.59	94.10	**97.00**
- [single sub-model 1]	1704	91.01	**91.85**	90.17	96.29
- [single sub-model 2]	1706	91.36	88.76	93.96	96.21
- [single sub-model 3]	1706	91.71	91.43	91.99	96.74
- [single sub-model 4]	1706	91.22	88.06	94.38	96.14
- [single sub-model 5]	1706	91.36	88.20	**94.52**	96.20

Supplementary Table S4: Minimum inhibitory concentrations (MIC) and minimum bactericidal concentrations (MBC) of selected AMP candidates following antimicrobial susceptibility testing (AST) *in vitro*. This is a supplementary table to Table 3. Candidate antimicrobial peptides were synthesized and purchased from Genscript. AST, and MIC/MBC determination was performed as outlined by the Clinical and Laboratory Standards Institute (CLSI) [4], with modification as recommended by Hancock [5]. Data is presented as the lowest effective peptide concentration range (µg/mL) observed in three independent experiments. LL37, human cathelicidin and a peptide from Tp0751 from *Treponema pallidum* were used as the positive and negative control peptides [6], respectively.

	***S. aureus*[a] ATCC 6538P** Gram-positive		***S. pyogenes*[b]** Gram-positive		***P. aeruginosa*[a] ATCC 10148** Gram-negative		***E. coli*[a] ATCC 9723H** Gram-negative		***E. coli*[c] ATCC 25922** Gram-negative		**MDR *E. coli*[d] (CPO-NDM)** Gram-negative	
**(µg/mL)**	**MIC**	**MBC**	**MIC**	**MBC**	**MIC**	**MBC**	**MIC**	**MBC**	**MIC**	**MBC**	**MIC**	**MBC**
**RaCa-1**	NI	NI	256	≥ 256	NI	NI	64 – 128	128 – 256	32 – 64	32 – 128	64 – 128	64 – 128
**RaCa-2**	2 – 4	2 – 4	64 – 128	64 – 128	64 – 128	128 – ≥256	8 – 16	8 – 16	4 – 16	4 – 16	4 – 16	4 – 16
**RaCa-3**	≥256	NI	128	128 – ≥ 256	64 – ≥256	128 – ≥256	16 – 32	16 – 32	8 – 16	8 – 16	16 – 32	16 – 64
**RaCa-4**	NI	NI	NI	NI	NI	NI	NI	NI	—	—	—	—
**RaCa-5**	NI	NI	NI	NI	NI	NI	NI	NI	NI	NI	NI	NI
**RaCa-6**	NI	NI	NI	NI	NI	NI	NI	NI	NI	NI	NI	NI
**RaCa-7**	≥ 256	NI	NI	NI	NI	NI	32 – 64	32 – 256	16 – 128	16 – 128	16 – 128	16 – 128
**RaCa-8**	NI	NI	NI	NI	NI	NI	NI	NI	NI	NI	NI	NI
**RaCa-9**	NI	NI	NI	NI	NI	NI	NI	NI	—	—	—	—
**RaCa-10**	NI	NI	NI	NI	NI	NI	NI	NI	NI	NI	NI	NI
**RaCa-11**	NI	NI	NI	NI	NI	NI	NI	NI	—	—	—	—
**LL37**	NI	NI	NI	NI	32 – ≥256	32 – ≥256	8 – 16	16 – 32	8 – 16	8 – 16	8 – 16	8 – 16
**Tp0751**	NI	NI	NI	NI	NI	NI	NI	NI	NI	NI	NI	NI

[a]Bacteria obtained and tested at the University of Victoria.
[b]Unknown strain; hospital isolate.
[c]ATCC quality control strain #25922 purchased from Cedarlane Laboratories (Burlington, Ontario, Canada).
[d]Clinical isolate obtained and tested at the British Columbia Centre for Disease Control.
NI, no inhibition observed *in vitro*.
'—' = not tested.
Abbreviations: *Staphylococcus aureus*, *Streptococcus pyogenes*, *Pseudomonas aeruginosa*, *Escherichia coli*; ATCC, American Type Culture Collection; CPO, carbapenemase-producing organism; MDR, multi-drug resistant; NDM, New-Delhi Metallo-beta-lactamase.

Supplementary Table S5: Predictions of Gaegurin 5 (GGN5) and its analogues by different AMP prediction tools. Antimicrobial activity data of GGN5 and its analogues were taken from the work by Won and con-workers [2]. The analogues were generated by truncating the parent peptide into shorter fragments and/or by amino acid substitutions. Prediction results of AMPlify, AMP Scanner Vr.2, and iAMPpred were listed for comparison.

Serial Number	Peptide Name[a]	Active?[b]	AMPlify		AMP Scanner Vr.2[c]		iAMPpred[d]			
			Score	Prediction	Score	Prediction	Score			Prediction
							ABP	AVP	AFP	
0	GGN5	✓	1.0000	AMP	1.0000	AMP	1.00	0.78	1.00	ABP, AVP, AFP
1	GGN5^{N9}		0.9940	AMP	0.9979	AMP	0.69	0.75	0.65	ABP, AVP, AFP
2	GGN5^{N11}		0.9819	AMP	0.9999	AMP	0.96	0.82	0.95	ABP, AVP, AFP
3	GGN5^{N12}		0.9934	AMP	1.0000	AMP	0.94	0.76	0.91	ABP, AVP, AFP
4	GGN5^{N13}	✓	0.9962	AMP	1.0000	AMP	0.95	0.77	0.92	ABP, AVP, AFP
5	GGN5$^{N11(3-13)}$	✓	0.9580	AMP	0.9793	AMP	0.99	0.74	0.95	ABP, AVP, AFP
6	GGN5$^{N13(2-14)}$		0.7552	AMP	0.9994	AMP	0.97	0.62	0.88	ABP, AVP, AFP
7	GGN5$^{N13(3-15)}$		0.9879	AMP	0.9968	AMP	0.91	0.38	0.73	ABP, AFP
8	GGN5$^{N13(4-16)}$		0.9944	AMP	0.9982	AMP	0.85	0.54	0.57	ABP, AVP, AFP
9	GGN5$^{N13(5-17)}$		0.8087	AMP	0.9995	AMP	0.94	0.63	0.71	ABP, AVP, AFP
10	G3K-GGN5^{N13}	✓	0.9944	AMP	1.0000	AMP	0.98	0.87	0.95	ABP, AVP, AFP
11	G3K/S10L-GGN5^{N13}	✓	0.9984	AMP	0.9999	AMP	0.98	0.90	0.94	ABP, AVP, AFP
12	F1A-GGN5^{N13}	✓	0.9948	AMP	0.9998	AMP	0.98	0.79	0.91	ABP, AVP, AFP
13	F1W-GGN5^{N13}	✓	0.8293	AMP	1.0000	AMP	0.93	0.81	0.77	ABP, AVP, AFP
14	F1W-GGN5^{N11}	✓	0.9356	AMP	1.0000	AMP	0.96	0.83	0.86	ABP, AVP, AFP
15	L2W-GGN5^{N11}	✓	0.9957	AMP	0.9999	AMP	0.85	0.86	0.69	ABP, AVP, AFP
16	G3W-GGN5^{N11}	✓	0.9971	AMP	1.0000	AMP	0.76	0.89	0.60	ABP, AVP, AFP
17	A4W-GGN5^{N11}	✓	0.9917	AMP	1.0000	AMP	0.85	0.88	0.70	ABP, AVP, AFP

18	L5W-GGN5^{N11}		0.9819	AMP	1.0000	AMP	0.85	0.86	0.69	ABP, AVP, AFP
19	F6W-GGN5^{N11}	✓	0.9888	AMP	1.0000	AMP	0.96	0.83	0.86	ABP, AVP, AFP
20	K7W-GGN5^{N11}		0.9909	AMP	0.9999	AMP	0.55	0.71	0.28	ABP, AVP
21	V8W-GGN5^{N11}	✓	0.9967	AMP	1.0000	AMP	0.86	0.88	0.78	ABP, AVP, AFP
22	A9W-GGN5^{N11}		0.9974	AMP	0.9999	AMP	0.85	0.88	0.70	ABP, AVP, AFP
23	S10W-GGN5^{N11}	✓	0.9810	AMP	0.9999	AMP	0.85	0.94	0.74	ABP, AVP, AFP
24	K11W-GGN5^{N11}		0.9824	AMP	0.9999	AMP	0.54	0.70	0.28	ABP, AVP
25	A4L-GGN5^{N11}	✓	0.9789	AMP	0.9999	AMP	0.98	0.81	0.97	ABP, AVP, AFP
26	A4K-GGN5^{N11}	✓	0.9957	AMP	1.0000	AMP	0.99	0.86	0.99	ABP, AVP, AFP
27	A4F-GGN5^{N11}	✓	0.8982	AMP	1.0000	AMP	0.84	0.85	0.88	ABP, AVP, AFP
28	V8L-GGN5^{N11}	✓	0.9936	AMP	0.9999	AMP	0.97	0.80	0.96	ABP, AVP, AFP
29	V8K-GGN5^{N11}	✓	0.9998	AMP	0.9999	AMP	0.99	0.86	0.98	ABP, AVP, AFP
30	V8F-GGN5^{N11}	✓	0.9943	AMP	0.9999	AMP	0.84	0.84	0.88	ABP, AVP, AFP
31	A4W/V8W-GGN5^{N11}	✓	0.9970	AMP	1.0000	AMP	0.86	0.91	0.30	ABP, AVP

[a]Peptides were taken from the work by Won and co-workers [2]; The right superscripts in the peptide name indicate the lengths and positions of truncation from N-terminals, while the left side characters indicate the positions and amino acids of substitution (e.g. GGN5$^{N11(3-13)}$ stands for a fragment of GGN5 from position 3 to 13 with length 11; G3K-GGN5^{N13} stands for a fragment of the first 13 residues from the N-terminal with a substitution of K for G at position 3); Please refer to the original paper for details.

[b]Whether the peptide shows antimicrobial activity against at least one of the ten bacterial species tested in the work: *Bacillus subtilis*, *Micrococcus luteus*, *Staphyloccus aureus*, *Staphylococcus epidermis*, *Escherichia coli*, *Shigella dysentariae*, *Salmonella typhimurium*, *Klebsiella pneumoniae*, *Proteus mirabilis*, and *Pseudomonas aeruginosa* [2].

[c]The “Orig. Production” model were chosen for AMP Scanner Vr.2 [1].

[d]iAMPpred predicts and classifies AMPs into ABPs (antibacterial peptides), AVPs (antiviral peptides), and AFPs (antifungal peptides) [7].

List of Abbreviations

Acc: Accuracy

ABP: Antibacterial peptide

AFP: Antifungal peptide

AMP: Antimicrobial peptide

AST: Antimicrobial susceptibility testing

AVP: Antiviral peptide

AUROC: Area under the receiver operating characteristic curve

Bi-LSTM: Bidirectional long short-term memory

CA: Context attention

Conv: Convolutional

LSTM: Long short-term memory

MBC: Minimum bactericidal concentration

MIC: Minimum inhibitory concentration

MHSDPA: Multi-head scaled dot-product attention

Sens: Sensitivity

Spec: Specificity

References

1. Veltri D, Kamath U, Shehu A. Deep learning improves antimicrobial peptide recognition. Bioinformatics. 2018;34:2740–7.
2. Won H-S, Jung S-J, Kim HE, Seo M-D, Lee B-J. Systematic peptide engineering and structural characterization to search for the shortest antimicrobial peptide analogue of gaegurin 5. J Biol Chem. 2004;279:14784–91.
3. Xiao X, Wang P, Lin W-Z, Jia J-H, Chou K-C. iAMP-2L: A two-level multi-label classifier for identifying antimicrobial peptides and their functional types. Anal Biochem. 2013;436:168–77.
4. Clinical and Laboratory Standards Institute. Methods for dilution antimicrobial susceptibility tests for bacteria that grow aerobically: approved standard. 2015.
5. Hancock REW. Modified MIC method for cationic antimicrobial peptides [Internet]. 1999 [cited 2017 Sep 22]. Available from: http://cmdr.ubc.ca/bobh/method/modified-mic-method-for-cationic-antimicrobial-peptides/
6. Helbing CC, Hammond SA, Jackman SH, Houston S, Warren RL, Cameron CE, et al. Antimicrobial peptides from Rana [Lithobates] catesbeiana: Gene structure and bioinformatic identification of novel forms from tadpoles. Sci Rep. 2019;9:1529.
7. Meher PK, Sahu TK, Saini V, Rao AR. Predicting antimicrobial peptides with improved accuracy by incorporating the compositional, physico-chemical and structural features into Chou's general PseAAC. Sci Rep. 2017;7:42362.
